# Virulence-Encoding Genes Conserved in *Salmonella* Isolated From Humans, Poultry, and Seafood

**DOI:** 10.1155/jotm/1139253

**Published:** 2025-07-02

**Authors:** Yemisi Olukemi Adesiji, Vijaya Kumar Deekshit, Rasheed A. Odunola, Indrani Karunasagar, Oluwafemi B. Daodu, Al-Mustapha Ahmad

**Affiliations:** ^1^Department of Medical Microbiology and Parasitology, College of Health Sciences, Ladoke Akintola University of Technology, Ogbomoso, Oyo, Nigeria; ^2^Department of Microbiology, K. S. Hegde Medical Academy, Faculty of Allied Health Services, Nitte University, Deralakatte, Mangalore, India; ^3^Department of Infectious Disease and Microbial Genomics, Nitte University Centre for Science Education and Research, Paneer Campus, Deralakatte, Mangaluru 575018, India; ^4^University Health Services, University of Ilorin, Ilorin, Kwara, Nigeria; ^5^Department of Veterinary Microbiology, Faculty of Veterinary Medicine, University of Ilorin, Ilorin, Kwara, Nigeria; ^6^Department of Veterinary Public Health and Preventive Medicine, Faculty of Veterinary Medicine, University of Ibadan, Ibadan, Oyo, Nigeria; ^7^Department of Food Hygiene and Environmental Health, Faculty of Veterinary Medicine, University of Helsinki, Helsinki, Finland

**Keywords:** humans, India, Nigeria, raw food, *Salmonella*, virulence genes

## Abstract

Diverse virulence genes encode for the Type III secretion system (T3SS) in bacteria. In *Salmonella*, these genes are located in the *Salmonella* pathogenicity Islands 1 and 2 (SPI-1 and SPI-2), and they facilitate bacterial invasion and replication within macrophages, contributing to the burden of nontyphoidal *Salmonella* infections. In this study, we investigated the prevalence of selected virulence-encoding genes in 30 laboratory stocks of *Salmonella enterica serovar Enteritidis* from Nigeria (16 isolates) and nonclinical sources comprising poultry and seafood from India (14 isolates). Analysis of PCR amplicons revealed that the genes *sseB*, *sseD, sseF, sse*T, and *invH* were conserved in all the isolates except for two isolates obtained from clams, which did not have the *sseD* and *sseF* genes. In addition, the *sseC* and *sseG* genes were absent from all the tested isolates. This study provides insights into the distribution of selected T3SS genes among *Salmonella* spp. isolated from clinical and raw animal food sources in Nigeria and India, respectively.

## 1. Introduction

*Salmonella* serovars are among the major causes of food-borne infections worldwide, including developing countries [1]. They cause salmonellosis, which affects both humans and animals and is mostly caused by nontyphoidal *Salmonella* serovars [2]. Salmonellosis is a One Health challenge, as it poses a significant zoonotic threat to humans globally. The pathogenicity of *Salmonella* is mediated by virulence genes located on *Salmonella* pathogenicity islands (SPIs) as well as on plasmids and on the chromosome [3]. The 24 currently known SPIs, especially SPI-1 and SPI-2, play a vital role in virulence in *Salmonella* serovars and encode the Type III secretion system (T3SS). The T3SS is capable of secreting proteins called effectors, thereby facilitating pathogen invasion, survival, and replication within the host cells [2–4].

The SPI-1 system enables the invasion of epithelial cells, while the SPI-2 system facilitates the survival and replication of intracellular bacteria within *Salmonella*-containing vacuoles [5, 6]. The virulence-encoding genes in SPI-2 encode a T3SS that codes for proteins that form the Type III secretion apparatus, serve as transcriptional regulators, and function as effectors that invade and usually result in host cell destruction [4, 7].

The true burden of food-borne invasive and gastroenteric *Salmonella* infections in humans is difficult to evaluate in developing countries such as Nigeria and India due to a lack of robust epidemiological (genomic) surveillance systems. The invasive form of nontyphoidal *Salmonella* (iNTS) has been reported in many countries in sub-Saharan Africa, including Nigeria, using whole-genome sequence-based data [8]. This short communication is the result of our screening for seven virulence genes present in SPI-1 (one gene) and SPI-2 (six genes) from our laboratory stock of previously characterized *Salmonella* isolates. Evaluation of these virulence-encoding genes aimed to improve the current knowledge of the T3SS in resource-limited settings.

## 2. Materials and Methods

### 2.1. Ethical Approval

The ethical clearance for this study was obtained from the ethical review committee of the University Health Services, University of Ilorin, Kwara State, Nigeria, with reference number UIL/UHS/71.

### 2.2. Sample Processing and Confirmation

Laboratory stocks of *Salmonella* spp. obtained from poultry (meat and fecal samples), seafood (clams), and human clinical sources were resuscitated using the ISO 6579-1 guidelines of 2017 [9] (Table 1). Briefly, the isolates were directly inoculated into Rappaport–Vassiliadis (RV) broth (Merck, Germany) and incubated for 18 ± 2 h at 37°C. All the overnight cultures were later plated on HiChrome-improved *Salmonella* agar (HiMedia, Laboratories Pvt. Ltd, India). Three biochemical tests were performed to characterize the *Salmonella* isolates: the urease test (urease medium), Simmons citrate test (Simmons citrate agar), and indole test (Kovac's reagent), following the manufacturer's instructions [10].

### 2.3. Molecular Characterization

DNA was extracted using the boil and spin method, a protocol described by Ahmed and Dablool in 2017 [11]. *Salmonella* isolates were genotypically confirmed by PCR using the housekeeping gene *invA*. This gene was selected because it is the gold-standard PCR target for the confirmation of *Salmonella*. The cycling conditions and primer sequences for the *invA* gene were as described by Jayaweera et al. [12].

### 2.4. Virulence Gene Detection

The extracted DNA of PCR-confirmed *Salmonella* isolates was then used for duplex PCR amplification to detect the presence of specific genetic elements. The genes targeted were *ssaT*, *sseB*, *sseF*, *sseG*, *sseD*, and *sseC* of SPI-2 and *invH* of SPI-1, as previously described [13]. The primer sequences, specific annealing temperature, and product size for each PCR reaction are provided in Table 2. The PCR reactions were carried out in a total volume of 30 μL, comprising 3 μL of 10x PCR buffer, 3 μL of 2 mM dNTPs, 1.5 μL of 10 μM primers (each primer), 0.5 μL of 5 U/μL Taq DNA polymerase, and 2 μL of 100 ng/μL DNA template. The reaction mixture was made up to 30 μL with the addition of 15.5 μL of water. The reaction mixture was then subjected to thermal cycling to amplify the target DNA sequence using the following amplification parameters: initial denaturation for 5 min at 95°C; 30 cycles of amplification consisting of denaturation for 15 s at 95°C, annealing for 30 s at 51°C and extension for 30 s at 72°C; and final extension for 7 min at 72°C. The PCR products were then separated on a 1.5% agarose gel (Fisher BioReagents, Geel, Belgium) and visualized using ethidium bromide (MP Biomedicals, Ohio, USA) staining on a gel documentation system (Bio-Rad, USA). *Salmonella enterica serovar Enteritidis* ATCC13076 was included in all sets of reactions as a positive control to validate the results.

## 3. Result

### 3.1. Prevalence of Virulence-Encoding Genes

**Figure 1:**
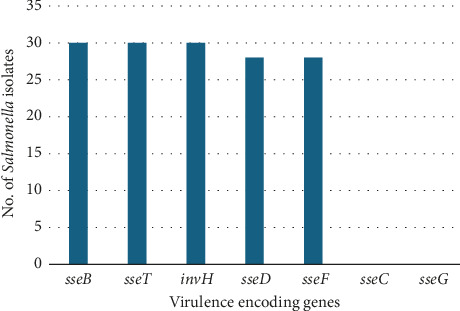
Distribution of virulence-encoding genes in *Salmonella* isolates of animal and human origin from Nigeria and India.

The 16 human clinical *Salmonella* isolates and the 14 nonhuman *Salmonella* isolates were confirmed as *Salmonella* by PCR amplification of the *invA* gene, which generated amplicons of 284 bp. The PCR for the virulence-encoding genes revealed that *sseT*, *sseB* (belonging to SPI-2), and *invH* (belonging to SPI-1) were conserved in all 30 *Salmonella* isolates. In addition, the detection rates for *sseD* and *sseF* were 93.3% (*n* = 28/30) for both genes. Notably, the *sseC* and the *sseG* genes were not detected in any of the tested isolates (Figure 1). The characteristics of the isolates included in this study are provided in Table 2.

## 4. Discussion

This study tested for the presence of selected virulence genes in *Salmonella* isolates. Our findings revealed that *sseB, sseT, sseD, sseF*, and *invH* were present in all the isolates. This could be because genes-encoding putative effector proteins of the T3SS of SPI-2 are required for bacterial invasion, virulence, and multiplication within macrophages [5]. Two clam isolates did not harbor the *sseD* and *sseF* genes. Many virulence genes can contribute to *Salmonella* pathogenicity, and the absence of one particular gene (as seen in the two clam isolates) could be compensated by the activities of other genes because of the redundancy known for the *Salmonella* genome. The transcriptional operon contains the cluster of most genes encoding components and effectors of the SPI-2 T3SS [6]. Moreover, *sseA* is the only reported chaperone for the SPI-2-encoded secretion apparatus proteins sse*B* and *sseD* [15]. No chaperones have been reported for any of the other SPI-2 effector genes, although *sse*A and *sse*B have been proposed to act as chaperones based on the sequence analysis [7]. Additionally, this study revealed that *sseD* and *sseF* genes were present in *Salmonella* isolates from humans and poultry but absent from seafood, suggesting that other virulence factors may contribute to the pathogenicity of this isolate in these animal species [15, 16].

In addition, *sseF* is one of the several genes required to confer bacteria survival by altering the trafficking of the *Salmonella*-containing vacuoles for SPI-2 [17]. Although not found in any of the isolates, the *sseG* gene was reported to be responsible for targeting *Salmonella* to the host Golgi network through poorly understood mechanisms [18]. In addition, studies have reported that *sse*G is not required for the translocation of effector proteins of SPI-2 [19, 20]. These observations indicated that *sseF* and *sseG* do not act as translocators for further substrate proteins in vivo and that their function is distinct from those of *sseB*, *sseC*, and *sseD*. Prior to this, it was reported that the effector proteins *sseC* and *sseD* predominantly localized in the bacterial cell membrane with the help of a functional SPI-2 T3SS [21].

The *invH* gene, which plays a role in SPI-1-mediated effector gene translocation, was present in all *Salmonella* isolates. It has previously been demonstrated that deletion of the *invH* gene in *S. enterica serovar Typhimurium* reduces its invasion efficiency by 70%–80% in vitro [22]. The protein encoded by the *invH* gene has been described to disrupt cytoskeletal and bacterial cell barriers, resulting in *Salmonella* invasion of the host gastrointestinal epithelium [23]. However, *S. enterica* serovar *Senftenberg* has been reported to elicit enteropathogenic infections in humans, despite the absence of many genes in the SPI-1 locus [24].

The genetic diversity of virulence-encoding genes in *Salmonella* requires further research to fully elucidate the functions and interactions between effector proteins (*sseA–sseG*) and other molecular markers [25–27]. In addition, these virulence-encoding genes were also harbored by enteropathogenic *Salmonella* spp. in poultry. Hence, routine hygiene measures include high-temperature cooking of poultry and clams and reducing the cross-contamination of ready-to-eat food. This study underscores the occurrence of *Salmonella* harboring virulence genes in humans, poultry, and seafood and highlights the need for One Health integrated surveillance. In addition, qualitative testing of food products is needed to guarantee their wholesomeness. The major limitations of this study are the small sample size and the fact that only a selection of genes in the T3SS was screened.

## Figures and Tables

**Table 1:** Isolates included in this study (*n* = 30).

Isolate ID	Serovar	Sample source	Year of isolation	Sample origin
Sal 1	Not typed	Clinical	2013	Nigeria
Sal 2	Not typed	Clinical	2013	Nigeria
Sal 3	Not typed	Clinical	2013	Nigeria
Sal 4	Not typed	Clinical	2013	Nigeria
Sal 5	Not typed	Clinical	2013	Nigeria
Sal 6	Not typed	Clinical	2013	Nigeria
Sal 7	Not typed	Clinical	2013	Nigeria
Sal 8	Not typed	Clinical	2013	Nigeria
Sal 9	Not typed	Clinical	2013	Nigeria
Sal 10	Not typed	Clinical	2013	Nigeria
Sal 11	Not typed	Clinical	2014	Nigeria
Sal 12	Not typed	Clinical	2013	Nigeria
Sal 13	Not typed	Clinical	2013	Nigeria
Sal 14	Not typed	Clinical	2013	Nigeria
Sal 15	Not typed	Clinical	2013	Nigeria
Sal 16	Not typed	Clinical	2013	Nigeria
Sal 17	*S. Weltevreden*	Poultry	2014	India
Sal 18	*S. Weltevreden*	Poultry	2014	India
Sal 19	*S. Weltevreden*	Poultry	2014	India
Sal 20	*S. Typhimurium*	Poultry	2014	India
Sal 21	*S. Typhimurium*	Poultry	2014	India
Sal 22	*S. Typhimurium*	Poultry	2014	India
Sal 23	*S. Typhimurium*	Seafood	2014	India
Sal 24	*S. Typhimurium*	Seafood	2014	India
Sal 25	*S. Typhimurium*	Clam	2014	India
Sal 26	*S. Weltevreden*	Seafood	2014	India
Sal 27	*S. Weltevreden*	Seafood	2014	India
Sal 28	*S. Weltevreden*	Seafood	2014	India
Sal 29	*S. Weltevreden*	Seafood	2014	India
Sal 30	*S. Typhimurium*	Clam	2014	India

**Table 2:** Primer sequences, target genes, and related details used in this study [13].

Target gene	Primer sequence	Melting temperature (^o^C)	Product size (bp)
*invH*	F: AGCAACTGGCCAACGCAAAT	57	153
	R: TGCAGTCTTTCATGGGCAGCAA		

*ssaT*	F: ATGGCACAACAGGTAAATGA	63	780
	R: TCATACAGATGGAAACCAGT		

*sseB*	F: 5′ATTGGATCCGGTGGAGATACCGTC	43	333
	R: TATGGATCCTGTTGTTAGGGTCGGG		

*sseC*	F: ATGAATCGAATTCACAGTAA	43	1455
	R: TTAAGCGCGATAGCCAGCTA		

*sseD*	F: ATGGAAGCGAGTAACGTAGC	41	588
	R: TTACCTCGTTAATGCCCGGA		

*sseE*	F: ATGGTGCAAGAAATAGAGCA	53	417
	R: TTAAAAACGTCGCTGGATAA		

*sseF*	F: ATGCGCAAATAATGGTTGAT	60	888
	R: TCAGGCGCGTTAACAGGACG		

*sseG*	F: ATGAAACCTGTTAGCCCAAA	60	690
	R: TTACTCCGGCGCACGTTGTT		

## Data Availability

Data sharing is not applicable to this article as no new data were created or analyzed in this study.
